# Standard coagulation assays alone are not sufficient to exclude surgically relevant rivaroxaban plasma concentrations

**DOI:** 10.1186/s13741-019-0128-9

**Published:** 2019-11-20

**Authors:** Alexander Kaserer, Andreas Schedler, Burkhardt Seifert, Donat R. Spahn, Jan-Dirk Studt, Philipp Stein

**Affiliations:** 1 Institute of Anaesthesiology, University and University Hospital Zurich, Raemistrasse 100, 8091 Zurich, Switzerland; 2 Department of Biostatistics at Epidemiology, Biostatistics and Prevention Institute, University of Zurich, Hirschengraben 84, 8001 Zurich, Switzerland; 3 Division of Medical Oncology and Haematology, University and University Hospital Zurich, Raemistrasse 100, 8091 Zurich, Switzerland

**Keywords:** Anticoagulation, Rivaroxaban, Coagulation assays, Surgery

## Abstract

**Background:**

While mainly larger hospitals have introduced routine anti-Xa assays for rivaroxaban (RXA), these are not readily available to smaller hospitals often relying on routine coagulation tests such as prothrombin time (PT) and activated partial thromboplastin time (aPTT).

The aim of our study was to investigate the effect of RXA plasma concentration on the standard coagulation tests PT (Quick test and INR) and aPTT in a large group of real-life patients. We further assessed whether normal results of these standard coagulation assays are sufficient to exclude surgically relevant RXA plasma concentration, defined as > 50 mcg/l.

**Methods:**

This retrospective study included all patients between 2012 and 2016 where anti-Xa (calibrated for RXA), PT (Quick test and INR), and/or aPTT were determined from the same sample. PT is expressed as Quick value (% of normal plasma pool). In total, 1027 measurements in 622 patients were eligible for analysis: 752 measurements of 505 patients for Quick/INR and 594 measurements of 417 patients for aPTT.

**Results:**

A moderate correlation of PT/Quick (Pearson's correlation coefficient − 0.59; *p* < 0.001), INR (Pearson's correlation coefficient 0.5; *p* < 0.001), and aPTT (Pearson's correlation coefficient 0.53; *p* < 0.001) with RXA plasma concentration was observed. However, in 50% of all samples with a normal PT/Quick, in 25% of all samples with a normal INR and in 80% of all samples with a normal aPTT residual RXA plasma concentration was surgically relevant.

**Conclusion:**

Although a moderate correlation of RXA plasma concentration with PT/Quick, INR, and aPTT was observed, standard coagulation assays are not sufficient to exclude surgically relevant RXA plasma concentrations.

## Background

Rivaroxaban (RXA) is a direct oral anticoagulant (DOAC) which inhibits factor Xa (Xa). It is widely used for thromboprophylaxis in orthopaedic surgery, prevention of systemic embolism in non-valvular atrial fibrillation, and treatment and secondary prevention of venous thromboembolism (Swissmedicinfo.ch 2017). In many patients admitted for elective and non-elective surgery, a surgically relevant residual RXA plasma concentration is observed, defined as > 50 mcg/l. This is especially important in an emergency setting (e.g., trauma) since it may affect patient management or be prohibitive for surgery.

For determining RXA plasma concentration, high-performance liquid chromatography-mass spectrometry (HPLC-MS) is considered the gold standard (Asmis et al. 2012). However, this method is time-consuming, expensive, and therefore not suitable for routine clinical use. In comparison, chromogenic anti-Xa assays calibrated for RXA are fast, affordable, and show a very good agreement with HPLC-MS (Studt et al. 2017). While most university hospitals or other large hospitals have introduced anti-Xa assays for RXA, these are not readily available to smaller hospitals often relying on routine coagulation tests such as prothrombin time (PT) and activated partial thromboplastin time (aPTT). In German-speaking countries, a plasma sample’s tissue factor-induced coagulation time is typically expressed as Quick value (%) in relation to that of a normal plasma pool; the longer the PT, the lower the Quick value (%).

The aim of our study was to investigate the effect of RXA plasma concentration as determined by anti-Xa assay on the standard coagulation tests PT (Quick test and INR) and aPTT. Further, we assessed whether normal values of these standard coagulation assays are sufficient to exclude surgically relevant RXA plasma concentration which was defined as > 50 mcg/l in a large group of real-life patients.

## Material and methods

This study was approved by the local ethics committee (Kantonale Ethikkomission Zurich, Switzerland, KEK-ZH-No: 2017-00164).

### Study design

This retrospective single-center study included all adult patients between 2012 and 2016 where anti-Xa (calibrated for RXA), PT (Quick test and INR), and/or aPTT were determined from the same plasma sample.

**Fig. 1:**
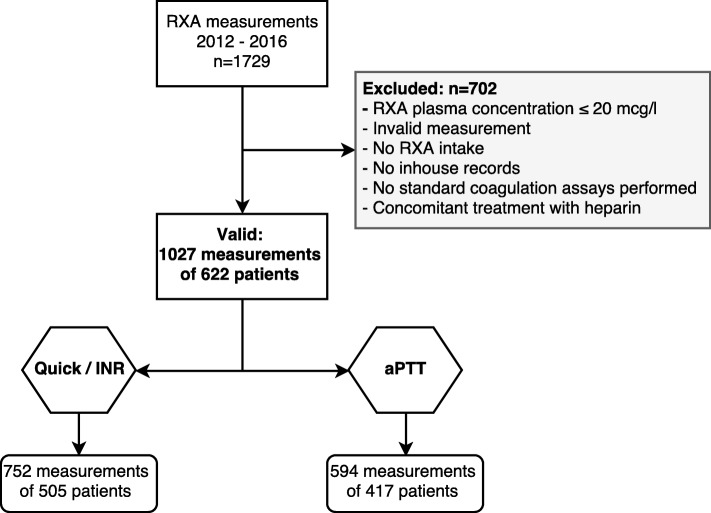
Flowchart showing the number of included and excluded patients for each standard coagulation assay. Not all patients had a concomitant Quick/INR and aPTT measurement, explaining the different counts in the Quick/INR and aPTT groups. RXA = rivaroxaban, INR=international normalized ratio, aPTT = activated partial thromboplastin time, Quick = prothrombin time expressed as % of the normal plasma pool

Patients with a RXA concentration below the assay’s limit of quantification (20 mcg/l), without documented RXA intake, without sufficient documentation, or with concomitant treatment by heparin or other anticoagulants were excluded. In total, 1027 measurements in 622 patients were eligible for investigation (Fig. 1).

### Study goals

Our study aimed to investigate the impact of anti-Xa determined RXA plasma concentration as determined by anti-Xa assay on the standard coagulation tests PT (Quick test and INR) and aPTT. Moreover, we assessed in a large group of real-life patients if normal values of these standard assays were sufficient to exclude a surgically relevant RXA plasma concentration.

### Sample preparation and coagulation assays

Venous blood was drawn into tubes containing 0.109 M sodium citrate (BD Vacutainer, Plymouth, UK). Samples were transported immediately to the ISO 17025 accredited hemostasis laboratory of the University Hospital Zürich where all coagulation assays were performed.

RXA plasma concentration was determined by chromogenic anti-Xa assay calibrated for RXA (Studt et al. 2017). During the daytime, the DiXaI assay (Hyphen Biomed, Neuville-sur-Oise, France) was used due to its insensitivity to heparin and at all other times a routine anti-Xa assay (Biophen Heparin LRT, Hyphen Biomed) with the same set of calibrators. Regular internal and external quality control was performed for both assays, and their agreement was confirmed repetitively.

In German-speaking countries, the tissue factor-induced coagulation time is traditionally expressed as Quick (%) instead of PT (s); therefore, PT is presented here as Quick (%) and INR. PT (Quick test) was determined using Innovin as thromboplastin reagent, and aPTT using the Actin FS reagent (both Siemens Healthcare, Marburg, Germany). Coagulometers were Siemens BCS XP and CS-5100. Normal values for standard coagulation assays were Quick > 70%, INR < 1.2, and aPTT 24–36 s.

### Variables and data collection

Medical records were reviewed of all patients where RXA plasma concentration was determined between 2012 and 2016. Age, sex, indication for anticoagulation, RXA dosage, body mass index (BMI), creatinine serum level, and glomerular filtration rate (GFR) according to CKD-EPI formula were extracted from the hospital’s database. The results of standard coagulation assays (Quick, INR, aPTT) originating from those samples in which RXA plasma concentration had been determined were extracted from the hospital’s laboratory information system. Data were transferred to a spreadsheet for evaluation (Excel 2016, Microsoft Corporation, Redmond, USA).

### Statistical analyses

Categorical data are reported as frequency (*n*) and percent (%) and numerical data as mean and standard deviation (SD) or median [IQR]. Due to the skew distribution of RXA plasma concentrations, INR and aPTT were logarithmically transformed. Correlation of RXA plasma concentrations with each of the standard coagulation assays was investigated using Pearson’s correlation coefficient. A receiver operating characteristic (ROC) curve analysis was performed calculating the area under the curve (AUC) for each standard coagulation assay. Statistical significance was set as a two-tailed *p* value of less than 0.05. All statistical analyses were performed with IBM SPSS Statistics (IBM SPSS Statistics v25.0., Armonk, NY: IBM Corp.).

## Results

### Patient characteristics

Patients were 58% male and 42% female, with a mean age of 69 ± 16 years. Renal function was mildly impaired with a mean GFR of 68 ± 27 ml/min and mean serum creatinine of 103 ± 63 mcmol/l. Indications for anticoagulation included atrial fibrillation, pulmonary embolism, deep vein thrombosis, and thromboprophylaxis. The most frequent RXA dose was 20 mg/day. The mean values of standard coagulation assays (PT/Quick, aPTT) were within the normal range (PT/Quick 70–120%; aPTT 24–36 s), and INR was slightly increased (Table 1).

**Table 1:** Overview

Age (years), mean ± SD		69 ±16
Sex male, *n* (%)		595 (58%)
Height (cm), mean ± SD		170 ± 10
Weight (kg), mean ± SD		76 ± 18
BMI (kg/m^2^), mean ± SD		26 ± 5
Serum creatinine (mmol/l), mean ± SD		103 ± 63
GFR CKD-EPI (ml/min), mean ± SD		68 ± 27
Indication for RXA, *n* (%)	Atrial fibrillation	574 (56%)
	Pulmonary embolism	128 (13%)
	Thrombosis	168 (16%)
	Prophylaxis	83 (8%)
	Other	74 (7%)
RXA dose (mg/day) (*n* = 1020) *n* (%)	10	69 (7%)
	15	200 (20%)
	20	712 (69%)
	30	38 (4%)
	40	1 (0%)
Quick (%), mean ± SD; median [IQR]		76 ± 23; 77 [74–79]
INR, mean ± SD, median [IQR]		1.3 ± 0.6; 1.2 [1.2–1.3]
aPTT (s), mean ± SD; median [IQR]		30 ± 12; 28 [28–29]

### PT/Quick and INR

**Fig. 2:**
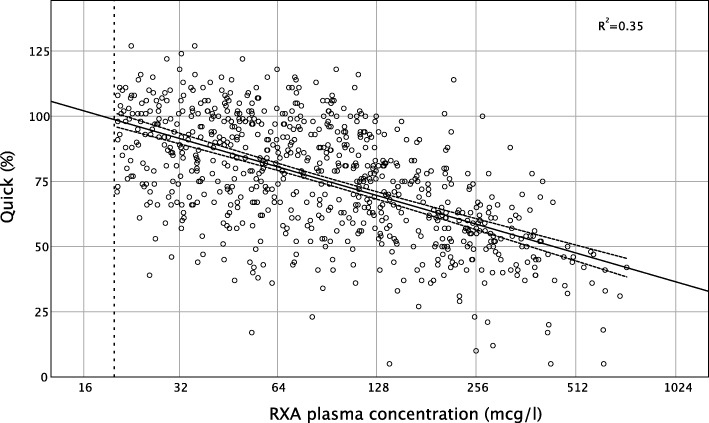
Correlation of rivaroxaban plasma concentration and Quick. A statistically significant correlation is observed (Pearson's correlation coefficient − 0.59, *p* < 0.001; *R*^2^ linear 0.35). Quick = prothrombin time expressed as % of normal plasma pool, RXA = rivaroxaban

Correlation of PT/Quick and RXA plasma concentration was moderate (Pearson's correlation coefficient − 0.59, *p* < 0.001; Fig. 2). Nevertheless, in 50% of all samples with a normal PT/Quick, the residual RXA plasma concentration was still elevated to a surgically relevant level > 50 mcg/l, up to a maximum of 407 mcg/l (AUC: 0.74, 95% CI 0.71 to 0.78, *p* < 0.001) (Table 2).

**Table 2:** Patients with normal standard coagulation assays but residual RXA level > 50 mcg/l

	RXA > 50 mcg/l		RXA plasma concentration mcg/l			
	*n*	%	Mean	SD	Min	Max
Quick > 70%	264	50%	77	57	20.4	407
INR < 1.2	133	25%	63	42	20.5	268
aPTT < 36 s	333	80%	100	79	20.4	437

**Fig. 3:**
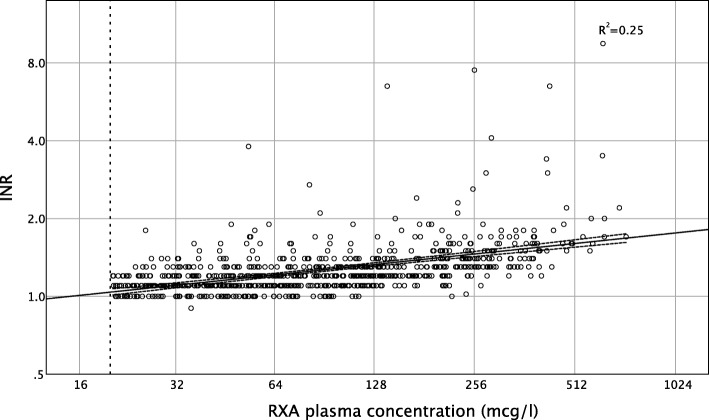
Correlation of rivaroxaban plasma concentration and INR. A statistically significant correlation is observed (Pearson's correlation coefficient 0.5, *p* < 0.001; *R*^2^ linear 0.25). INR = international normalized ratio, RXA = rivaroxaban

Similarly, the correlation of INR with RXA plasma concentration was only moderate (Pearson's correlation coefficient 0.5, *p* < 0.001; Fig. 3). Again, 25% of all patients with INR < 1.2 had a surgically relevant residual RXA plasma concentration up to a maximum of 268 mcg/l (AUC 0.74, 95% CI 0.70 to 0.77, *p* < 0.001) (Table 2).

### aPTT

**Fig. 4:**
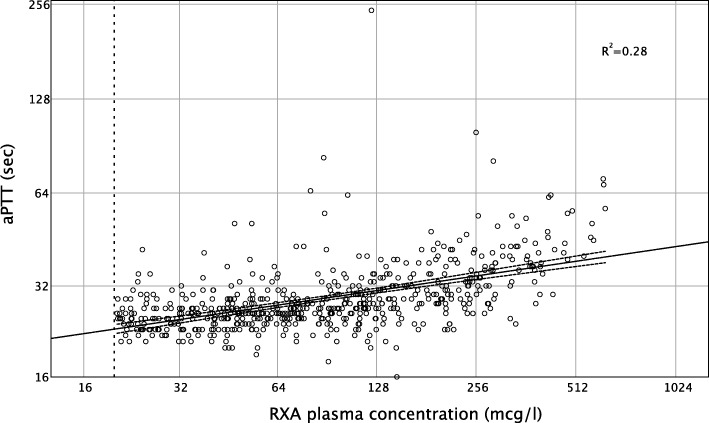
Correlation of rivaroxaban plasma concentration and aPTT. A statistically significant correlation is observed (Pearson's correlation coefficient 0.53, *p* < 0.001; *R*^2^ linear 0.28). aPTT = activated partial thromboplastin time, RXA = rivaroxaban

APTT showed as well a moderate correlation with RXA plasma concentration (Pearson's correlation coefficient 0.53, *p* < 0.001; Fig. 4). As many as 80% of all patients with a normal aPTT (< 36 s) had a surgically relevant RXA plasma concentration up to a maximum of 437 mcg/l (AUC 0.75, 95% CI 0.71 to 0.79, *p* < 0.001) (Table 2).

## Discussion

An increasing number of patients admitted to the emergency department are anticoagulated with DOACs such as RXA. If such information is lacking—e.g., because of an unconscious patient—significant bleeding during invasive procedures or thrombolysis may be the consequence. Furthermore, factors were identified such as renal insufficiency or amiodarone co-medication that may result in a higher-than-expected residual RXA concentration even if standard preoperative interruption intervals are observed (Kaserer et al. 2018). Fast and reliable quantification of a DOAC’s plasma concentration is therefore crucial for the clinical management. While many universities or other large hospitals have introduced routine anti-Xa assays for RXA, smaller hospitals often have to rely on standard coagulation assays such as PT/Quick, INR, or aPTT. Although we observed a statistically significant (but moderate) correlation of the results of these standard assays with RXA plasma concentration, these were not sufficient to exclude a surgically relevant residual RXA level.

In line with our results, a close correlation of anti-Xa activity, PT, and aPTT has been observed previously in healthy individuals (Turkoglu 2015). APTT showed a slight to moderate prolongation depending on the RXA concentration (Samama et al. 2010). In an in vitro study simulating RXA peak plasma concentrations in healthy volunteers, the aPTT was prolonged two-fold by RXA peak plasma levels of 389 ± 106 mcg/l to 617 ± 149 mcg/l (Hillarp et al. 2014). Moreover, Ikeda and Tachibana showed in patients receiving RXA for atrial fibrillation that aPTT tends to be prolonged (Ikeda and Tachibana 2016). The correlation of aPTT and RXA plasma level does not only depend on the latter since there is considerable variability among laboratories and various aPTT reagents (Samuelson et al. 2017; Samama et al. 2013). Although the reagent used in our laboratory (Actin FS, Siemens Healthcare) is comparatively sensitive to RXA (Samama et al. 2013), aPTT was not prolonged in 80% of patients with a residual RXA level > 50 mcg/l.

The effect of RXA plasma level on aPTT was weaker than for PT/Quick and INR. It was shown, that RXA prolongs PT/Quick in a linear and concentration-dependent manner (Samama et al. 2010). Baglin et al. demonstrated that normal PT cannot exclude an anticoagulant effect of DOAC, but can indicate a subtherapeutic plasma level (Baglin 2013). Results vary according to the thromboplastin reagent (Samama et al. 2010; Dale et al. 2014). Innovin (Siemens Healtcare), which is used in our laboratory, has an intermediate sensitivity towards RXA as compared with other reagents, e.g., recombiplastin, neoplastin, or neoplastin plus (Samama et al. 2010). The RXA plasma concentration required to prolong the PT two-fold is 301 mcg/l using neoplastin plus compared with 700 mcg/l using Innovin (Samama et al. 2010; Perzborn et al. 2005). A systematic review of 49 articles showed that the prolongation of PT depends on the plasma concentration of RXA, but that the correlation was weaker above 50–100 mcg/l (Samuelson et al. 2017).

Although a significantly elevated INR was noted in patients taking DOAC (Ofek et al. 2017), conversion of PT to INR increased the variability and resulted in reduced RXA responsiveness (Siegal and Konkle 2014). This is not surprising since INR was designed for the monitoring of anticoagulation with vitamin K antagonists, with a focus on the usual target range of INR 2.0–3.0. Interestingly, only 25% of our patients with a normal INR (< 1.2) had a RXA plasma concentration above 50 mcg/l which is the lowest proportion compared to patients with normal aPTT and/or PT/Quick.

In line with the findings of our study, the Guidance from the British Committee for Standards in Haematology by Kitchen et al. concluded that PT and aPTT cannot be used to quantify RXA plasma concentration. At most, these assays - with some but not all reagents – may permit a crude estimation of the intensity of anticoagulation (Kitchen et al. 2014). Anti-Xa chromogenic assays should be used instead to determine RXA plasma concentration (Kitchen et al. 2014; Adcock and Gosselin 2015).

### Limitations

Data of our study were collected retrospectively. Nevertheless, documentation and data collection followed Good Clinical Practice guidelines, and we assume that the data quality is high.

## Conclusion

Although a moderate correlation of RXA plasma concentration with PT/Quick, INR and aPTT was observed, standard coagulation assays alone are not sufficient to exclude a surgically relevant RXA plasma concentration. Chromogenic anti-Xa assays should be used in patients with suspected RXA intake.

## Data Availability

All data generated or analysed during this study are included in this published article.

## Abbreviations

aPTT: Activated partial thromboplastin time
AUC: Area under the concentration-time curve
BMI: Body mass index
DOAC: Direct oral anticoagulants
GFR: Estimated glomerular filtration rate according to the CKD-EPI formula
INR: International normalized ratio
PT: Prothrombin time
ROC: Receiver operating characteristic
RXA: Rivaroxaban
